# We need a new concept: from complementary examination to a source of value

**DOI:** 10.1590/0100-3984.2019.52.4e2

**Published:** 2019

**Authors:** Leonardo Vedolin

**Affiliations:** 1 Radiologist and Director of Imaging at DASA, São Paulo, SP, Brazil. Email: leonardo.vedolin@dasa.com.br.

The use of imaging technology in value-based medicine is a recurring theme in associative and academic debates, as well as in the health care market decision-making process. Its main driver is the premise that everyone involved in the value chain should propose solutions that optimize the improvement of health outcomes, in order to align the expectations of all those involved in the journey. In practice, the cost/waste of these technologies prevails as the “villain” in the equation.

Proving value in radiology and diagnostic imaging is not a trivial task. Although the concept of value-based medicine is being extensively discussed, the form and intensity of radiologist adherence to the concept in practice has not been widely studied. Some speak of “gaining a better understanding of patients”, whereas others mention “being better able to quantify their work performance”. The feeling is that our specialty is at a crossroads on our way to our final destination, with no strength (or focus) to choose the next road to go down.

Could using a modern imaging technology to detect a disease for which there is no reliable biomarker be an example of value-based medicine? Does optimizing the use of this technology to detect the greatest number of patients with such a disease and exclude other diagnostic possibilities not add value to the chain of diagnosis and treatment of the disease?

In an article published in the previous issue of **Radiologia Brasileira**, Abreu Junior et al.^(^^1^^)^ demonstrated that the use of diffusion-weighted magnetic resonance imaging was able to detect alterations related to transient global amnesia in a sample of patients treated in the emergency department of a tertiary referral hospital, and that optimization of the protocol (adjusting the b value in the sequence) increased the sensitivity of the method in the patients evaluated. Although it was not the objective of the study, it would be interesting to know to what degree this image-based information modified the preliminary clinical diagnosis; reduced the cost by decreasing the number of unnecessary examinations; shortened hospital stays; and improved the system by establishing insights into the prognosis and recurrence of similar events.

Although the use of diffusion-weighted magnetic resonance imaging in transient global amnesia is not a recent example of the application of the method in neurology, it can be a powerful tool to demonstrate the “value” of an extraordinary technology, in addition to its traditional (restricted) role as a diagnostic tool. The moment for demonstrating that radiology is much more than a set of complementary tests that facilitate the diagnosis has passed. There is an urgent need for a conceptual evolution regarding the role of radiology (and radiologists) in value-based medicine.
